# β-Thalassemia Mutation At Codon 37 (Tgg>>Tga) Detected In A Turkish Family

**DOI:** 10.4274/Tjh-2013.0073

**Published:** 2013-09-05

**Authors:** Derya Güleç, Sibel Bilgili, Nuriye Uzuncan, Giray Bozkaya, Nur Soyer, Baysal Karaca

**Affiliations:** 1 İzmir Bozyaka Eductaion Research and Hospital, Biochemitry Department, İzmir

## TO THE EDITOR

The β-globin gene mutation at codon 37 [TGG (Trp)→TGA (stop codon)] gives rise to a β0-thalassemia that was described first by Boehm et al. in 1986 in a Saudi Arabian family [1]. Thereafter, other nonsense codon 37 mutations have been reported [1,2,3,4]. Another mutation at codon 37 (TGG/TAG; tryptophan→stop codon) has also been reported previously [5,6]. 

Premature stop of translation results in a truncated protein and usually the phenotype of β-thalassemia major in homozygous individuals. 

We have found an example of the nonsense codon (TGG→TGA; Trp→Stop) in a Turkish family. We report 3 cases with 1 homozygous and 2 heterozygous mutations at codon 37 causing a premature stop codon. 

Human fetal hemoglobin is present in vivo as both an acetylated F1 (ααγγacetyl) form by the presence of acetyl groups at the NH2 termini of the γ chains and a nonacetylated F0 (ααγγ) form. The fraction of the total fetal hemoglobin in acetylated form (F1) is about 10%, a value similar to that reported previously for cord erythrocytes and mostly in newborns [7,8]. 

A 37-year-old female patient (case 1) was admitted to our hospital with symptoms of anemia and repeated blood transfusion dependence once a year. Her red blood cell count (RBC) was 4.34x1012/L, hemoglobin (Hb) was 97 g/L 9 g/L, mean corpuscular volume (MCV) was 69.1 fL (<80 fL), and mean corpuscular hemoglobin (MCH) was 22.4 pg (<27 pg ). Her hemoglobin subtypes were quantified by high-performance liquid chromatography and HbA was 0% (70.0%-95%), HbF0 was 89.0% (<1.5.0%), HbF1 was 10.0%, and HbA2 was 1.0% (<3.5%). The blood smear showed microcytosis, hypochromia, teardrop cells, and target cells. The patient’s family was originally from the eastern region of Turkey and we were not able to take her parents’ blood samples. Consanguinity is not known to be the case in this family. Her 1-year-old son’s (case 2) and her sister’s (case 3) hematological parameters are given with the patient’s in Table 1.

The β-globin genomic DNA was analyzed after receiving informed consent. The β-globin regions of interest were amplified from isolated DNA in a single multiplex polymerase chain reaction and DNA sequencing analyses were done using an ABI 310 sequencer (Applied Biosystems, Foster City, CA, USA). Direct forward and reverse sequencing of the genes revealed that case 1 was homozygous and the other cases were heterozygous for the codon 37 (TGG→TGA) mutation. This mutation results in the production of a premature termination codon (tryptophan→stop codon) and gives rise to β0-thalassemia. Informed consent was obtained.

Prevention of β-thalassemia requires knowledge of the molecular spectrum occurring in the population at risk. This knowledge is particularly necessary when prevention control is applied to a multiethnic population. The frequency of this nonsense codon 37 mutation in the Turkish population is not known. 

## CONFLICT OF INTEREST STATEMENT

The authors of this paper have no conflicts of interest, including specific financial interests, relationships, and/ or affiliations relevant to the subject matter or materials included. 

## Figures and Tables

**Table 1:**
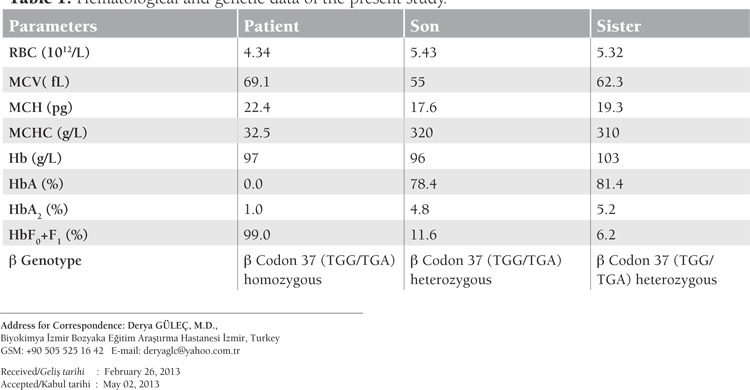
Hematological and genetic data of the present study
